# Isolation of the mustard Napin protein *Allergen Sin a 1* and characterisation of its antifungal activity

**DOI:** 10.1016/j.bbrep.2022.101208

**Published:** 2022-01-15

**Authors:** Giulia Mignone, Laila N. Shwaiki, Elke K. Arendt, Aidan Coffey

**Affiliations:** a Department of Biological Sciences, Munster Technological University, Cork, T12P928, Ireland; b School of Food and Nutritional Sciences, University College, Cork, T12K8AF, Ireland; c APC Microbiome Ireland, University College, Cork, T12K8AF, Ireland

**Keywords:** Mustard, Antifungal protein, Napin proteins, *Allergen Sin a 1*

## Abstract

Proteins and peptides belonging to the plant immune system can possess natural antibacterial, antifungal and antiviral properties. Due to their broad range of activity and stability, they represent promising novel alternatives to commonly used antifungal agents to fight the emergence of resistant strains. An isolation protocol was optimised to target proteins found in plants’ defence system, and it was applied to white mustard (*Brassica hirta*) seeds. Firstly, a ∼14 kDa protein with activity against *S. cerevisiae* was extracted and purified; secondly, the protein was identified as the mustard Napin protein named *Allergen Sin a 1*. Napin is the name given to seed storage (2S) albumin proteins belonging to the *Brassicaceae* family. While several Napins have been described for their antimicrobial potential, *Sin a* 1 has been mainly studied for its allergenic properties. The antimicrobial activity of *Sin a 1* is described and characterised for the first time in this study; it possesses antifungal and antiyeast *in vitro* activity, but no antibacterial activity was recorded. The yeasts *Zygosaccharomyces bailii* Sa 1403 and *Saccharomyces cerevisiae* DSM 70449 along with the filamentous fungi *Fusarium culmorum* FST 4.05 were amongst the most senstitive strains to *Sin a 1* (MICs range 3–6 μM). The antimicrobial mechanism of membrane permeabilisation was detected, and in general, the antifungal activity of *Sin a 1* seemed to be expressed in a dose-dependent manner. Data collected confirmed *Sin a 1* to be a stable and compact protein, as it displayed resistance to α-chymotrypsin digestion, heat denaturation and insensitivity to pH variations and the presence of salts. In addition, the protein did not show cytotoxicity towards mammalian cells.

## Highlights

• Simple purification of an antiyeast protein from white mustard seeds.
• Identification of the protein as the mustard Napin also classified as *Allergen Sin a 1*.
• Description of *Sin a 1* antimicrobial spectrum and mode of actions against yeasts.

## 1 Introduction

In recent years, a rapid rise of fungal (and yeast) strains resistant to the commonly used antifungal agents has been recorded. These microbes can present a worldwide threat to human health, agricultural production and food industries [1]. Alternatives to the commercially available antifungal molecules are scarce; thus, it is crucial to discover and characterise new products to fight the emergence of these troublesome microorganisms.

Natural antifungal compounds are present in many animal, plant, bacterial and fungal organisms that have evolved to produce biologically active substances to respond to pathogenic environmental fungi. The designation AMPs (Antimicrobial Peptides) is often associated with this type of molecule. Kingdom *Plantae* is an excellent source of biologically active antimicrobial proteins and peptides showing divergent genetic origins and structures; however, plant AMPs are generally characterised by low molecular weight and amphiphilic and cationic properties [2]. These molecules mainly belong to the plant innate immunity system, and they are often found accumulated in different plant tissues; therefore, they represent an ideal target for the investigation of new antifungal and antiyeast compounds [3]. However, the isolation of these molecules from vegetable materials can be complex. Protocols for plant protein isolation must be developed while considering the presence of rigid cellulose cell walls and vacuoles containing secondary plant products, organic acids and proteinases. Specific extraction methods are essential to protect proteins from the degradation activity of components leaked after cell wall disruption and consequent vacuole breakage [4]. Although plant AMPs can be synthesised in different tissues (leaves, flowers and roots) [5], seeds have usually been the plant organ of choice to isolate novel AMPs due to their relative higher abundance [6].

The plant family *Brassicaceae* (or *Cruciferae*) includes valuable crops such as cabbage, broccoli, cauliflower, kale, Brussels sprouts, turnip, rocket salad, watercress, radish, horseradish, wasabi, rapeseed and white, Indian and black mustard. Several of these plants have been investigated for their antimicrobial, antioxidant and anticancer properties [7]. In particular, antifungal proteins and peptides have been isolated from the seeds of radish [8], broccoli [9] and rapeseed plants [10]. White mustard (*Sinapis alba* (L) or *Brassica hirta*) is the most used mustard species in Europe, and its seeds are primarily used as a spice and for their high oil content [11]. Mustard seeds are known for possessing antimicrobial properties; however, there is a lack of studies on the antimicrobial activity of purified mustard proteins [12].

The main goal of this work was to isolate, identify and characterise the activity of peptides or small proteins with antifungal potential from white mustard seeds. Moreover, data collected on the toxicity and the stability of these compounds helped evaluate potential biotechnological applications.

## 2 Material and Methods

### 2.1 Selective isolation of the antifungal protein

The isolation of the antifungal protein from white mustard (*B. hirta*) seeds was achieved following the indications by *Koo* et al., 1997 with few significant modifications. Briefly, 50 g of dry white mustard seeds (Fruit Hill Farm/Veyranno Ltd T/A, Bantry, Ireland) were homogenised in a coffee grinder. The resulting flour was resuspended in cold buffer (15 mM of Na_2_HPO_4_, 10 mM of NaH_2_PO_4_, 100 mM of KCl, 1 mM of EDTA and 1 mM thiourea) in a 1:10 (w/v) ratio. Unless stated otherwise, all chemicals and media used in this work were purchased from Sigma Aldrich (MO, USA). The sample was extracted under gentle stirring for 3 h at 4 °C; subsequently, solid materials were removed by centrifugation (45 min at 7000 g) and filtration (Whatman filter paper Grade 595, Sigma Aldrich, MO, USA). Solid ammonium sulphate (NH_4_)_2_SO_4_ was added to the solution until 30% of relative saturation, and the sample was stirred at 4 °C. After 24 h, the resulting precipitate was removed by centrifugation. The supernatant was adjusted again with solid ammonium sulphate to reach 70% of relative saturation; the final precipitate formed overnight (4 °C) was collected by centrifugation and re-dissolved in 75 ml of dH_2_O. The solution was then heated at 80 °C for 15 min, and heat-denatured protein precipitates were removed by centrifugation. The supernatant was extensively dialysed against distilled water in dialysis tubing with a molecular mass cut-off of 2,000 Da (Sigma Aldrich, MO, USA). After three days, the proteins and peptides solution obtained was clarified again through filtration and collected. Protein purification was achieved after one cycle of cation-exchange chromatography in an ÄKTA-start system (GE Healthcare, Uppsala, Sweden). The proteins and peptides solution was adjusted to 10 mM phosphate buffer (pH 6.0) and 25 mM NaCl and loaded on a 5 ml HiPrep SP HP cation-exchange column (GE Healthcare, Uppsala, Sweden). Fractions were eluted with a linear gradient of 0–100% elution buffer (10 mM PBS and 520 mM NaCl, pH 6) in phosphate buffer (10 mM PBS and 25 mM NaCl, pH 6) at a flow rate of 5 ml/min within 60 min. The different peaks (5 ml fraction) found in the eluted fraction were de-salted (Slide-A-Lyzer MINI Devices 3.5 kDa MWCO, Thermo Scientific, MA, USA), filter-sterilised (pore size: 45 μm) and tested for their antifungal activity against the yeast *Saccharomyces cerevisiae* DSM 70449. Active antiyeast fractions were combined and dried in a freeze dryer (benchtop K VirTis, SP Industries, MO, USA). The resulting powder was dissolved in distilled water to reach 2 mg/ml concentration, filter-sterilised (pore size: 45 μm) and stored at −20 °C for further analysis. The protein concentration was determined by a BCA (Bicinchoninic Acid) assay (QuantiPro BCA Assay Kit, Sigma Aldrich, MO, USA) for total protein quantification.

### 2.2 Gel electrophoresis

Purified protein samples for the Sodium Dodecyl Sulphate Polyacrylamide Gel Electrophoresis (SDS-PAGE) assay were prepared in a dilution 1:2 with 2x SDS gel loading buffer (in the absence and the presence of a reducing agent) and boiled for 10 min at 95 °C (Applied Biosystems Thermal Cycler, Thermo Scientific, MA, USA). One hundred mM of dithiothreitol (DTT) (Thermo Scientific, MA, USA) was used as reducing agent. The gel was prepared using TGX FastCast Acrylamide Starter Kit, 12% (Bio-Rad Laboratories, CA, USA) and following the manufacturer's instructions. After the run, protein bands were stained with EZBlue Gel Staining Reagent (Sigma Aldrich, MO, USA).

### 2.3 Protein identification

A “*de novo* protein sequencing analysis” was carried out commercially (Creative Proteomics, New York, USA) to identify the protein's primary structure. Firstly, non-reduced SDS-gel bands were digested by six different enzymes: trypsin, chymotrypsin, Glu-C, Arg-C, Lys-N, and pepsin; subsequently, the amino acidic sequence of each peptide fragment was determinate by nanoscale liquid chromatography coupled with tandem mass spectrometry (nano LC-MS/MS). Finally, data from peptide mapping analysis were explored with the software PEAKS STUDIO Desktop Version 8.5 to identify the whole protein sequence. The protein amino acid sequence was subjected to a BLASTp search on the NCBI database (https://blast.ncbi.nlm.nih.gov/Blast.cgi?PAGE=Proteins) to determine the percentage of identity with existing sequences. The amino acid sequences of the closest related protein were downloaded in FASTA format and aligned with the help of the online tool Clustal Omega – Multiple Sequence Alignment (https://www.ebi.ac.uk/Tools/msa/clustalo/).

### 2.4 Minimum inhibitory concentration

The antimicrobial potency of the purified protein was investigated, calculating the minimum inhibitory concentration (MIC) for several bacterial and fungal strains (Table 1). All the microbial strains used were either present in the MTU collection or purchased from the Leibniz Institute DSMZ collection (Deutsche Sammlung von Mikroorganismen und Zellkulturen GmbH, Germany). The MIC value for each strain was found using broth dilution methods where the growth of each strain, exposed to serially diluted protein concentration, was monitored by a microtiter plate reader (Multiskan FC Microplate Photometer, Thermo Scientific, MA, USA) in a flat bottom 96-well plate. Tests were done on two protein concentration series ranging from 1000 μg/ml to 31.25 μg/ml and from 800 μg/ml to 25 μg/ml. Water without protein was used as a control. The MIC was determined as the lowest concentration of protein that prevented the visible growth of the microorganism after the incubation step. MIC data were obtained as μg/ml and converted to μM using the theoretical mass value of the protein.

**Table 1:** *Sin a 1* MIC range against yeasts, moulds and bacteria.

Strains	MIC range	
	μg/ml	μM
*Kluyveromyces lactis* ATCC 56498	200–250	13–16
*Debaryomyces hansenii* CBS 2334	150–200	9–13
*Zygosaccharomyces bailii* Sa 1403	50–100	3–6
*Zygosaccharomyces rouxii* ATCC 14679	400–500	25–31
*Saccharomyces cerevisiae* DSM 70449	100	6
*Saccharomyces cerevisiae* NCYC 77	100–200	6–13
*Saccharomyces cerevisiae* MTU 01P	1000	63
*Candida albicans* CUH 001	1000	63
*Fusarium culmorum* FST 4.05	50–100	3–6
*Aspergillus fumigatus* DSM 15966	1000	63
*Escherichia coli* ATCC 25922	No inhibition	
*Micrococcus luteus* CIT3	No inhibition	

#### 2.4.1 Antibacterial assay

The antibacterial activity was assessed following the protocol by Thery [9] against strains *Escherichia coli* ATCC 25922 and *Micrococcus luteus* CIT3. Bacteria were cultured on Mueller-Hinton agar for 24 h at 37 °C. One colony was transferred to tryptic soy broth for 2 h and diluted with PBS until the OD of the media reached McFarland 1 (DEN-1 McFarland densitometer, Biosan Limited, UK). Mueller-Hinton broth was used as the growth media, and 2.5 μl of the bacterial solution was added to each well. The OD at 600 nm was monitored continuously for 24 h at 37 °C.

#### 2.4.2 Antifungal assay

The activity against filamentous fungi was assessed with an inhibition of conidial germination assay as described by Thery [9]. Tests were conducted on *Aspergillus fumigatus* DSM 15966 and *Fusarium culmorum* FST 4.05 strains. Moulds were cultured on potato glucose agar for 5/7 days at 30 °C; afterwards, fungal spores were collected using 10 ml of dH_2_O and a cell strainer. The final inoculum was prepared in half-strength potato dextrose broth (½ PDB) at a 10^5^ spores/ml concentration. One hundred microliters of spore solution were added to each well in conjunction with 100 μl of protein at different dilutions; the spore concentration was calculated using a haemocytometer (Improved Neubauer Counting Chamber, Sigma-Aldrich, MO, USA). Fungal growth was monitored at 28 °C for 48 h, and absorbance was followed by spectrophotometry at 600 nm at 2 h intervals.

#### 2.4.3 Antiyeast assay

The purified protein antiyeast potency was evaluated on 8 yeast strains; *Kluyveromyces lactis* ATCC56498, *Zygosaccharomyces bailii* Sa1403, *Zygosaccharomyces rouxii* ATCC14679, *Debaryomyces hansenii* CBS2334, *Saccharomyces cerevisiae*: DSM 70449, *Saccharomyces cerevisiae*: NCYC 77, *Saccharomyces cerevisiae*: MTU 01P and *Candida albicans* CUH 001. Antiyeast assays were carried out following the guidelines of the “EUCAST Definitive Document EDef 7.1: method for the determination of broth dilution MICs of antifungal agents for fermentative yeasts” [13]. Briefly, 100 μl of the solution under investigation were mixed with 100 μl of a 5 × 10^4^ cells/ml yeast inoculum in Sabouraud Dextrose (SD) broth; the inoculum was prepared from an overnight culture (Malt Extract broth). The yeasts’ growth was monitored, measuring the optical density (600 nm) at 2 h intervals, for 48 h at 30 °C.

### 2.5 Colony count assay

A colony count assay was performed to determine the killing efficiency of the protein against the strain *S. cerevisiae* DSM 70449. Briefly, a yeast suspension of 10^4^ cells/ml was prepared in conjunction with 25, 50, 100, 200 and 400 μg/ml of the antifungal protein. One hundred microliters of each suspension were spread every hour onto SD agar plates; this was repeated every hour for a total period of 6 h. Colonies were counted after incubating plates for 48 h at 30 °C.

### 2.6 Total nucleotide leakage

The total nucleotide leakage of the type strain *S. cerevisiae* DSM 70449 resulting from the activity of different concentrations of the antifungal protein was calculated according to Shwaiki [14]. Briefly, a 10^6^ cells/ml yeast suspension was prepared, washed twice in phosphate saline buffer (PBS) and incubated at 30 °C for 5 h with protein concentrations of 25, 50, 100, 200 and 400 μg/ml. Afterwards, yeast cells were removed via filtration through a 0.22 μm filter, and the OD 260 nm of the filtrate was recorded (M 501 UV-VIS, Spectronic Camspec Ltd, Leeds, UK). Triton X-100 (0.1%) (Sigma Aldrich, MO, USA) and water were used as positive and negative controls, respectively.

### 2.7 Membrane permeabilisation assay

The protein's ability to permeabilise the membrane of the yeast *S. cerevisiae* DSM 70449 was studied as a possible fungicidal mechanism of action. Propidium iodide (PI) (Sigma Aldrich, MO, USA) is a fluorescent dye that can bind nucleic acids of cells; since it is excluded from viable cells, the bond occurs only when the cell membrane has been permeabilised. The protocol by Canelli [15] was followed with some modifications. Briefly, a 10^6^ cells/ml suspension was prepared in PBS from an overnight culture in SD broth and exposed to 50, 100, 200 and 400 μg/ml of protein. Yeast cells treated with 0.1% Triton-X and water were used as positive and negative controls, respectively. After 5 h incubation at 30 °C, the cells were washed in PBS twice. Subsequently, samples were incubated in dark conditions for 5 min at room temperature in conjunction with 200 μl of PI (6 μM). Then, treated cells were washed twice again with PBS, and the pellets were resuspended in 250 μl of PBS, and 4 μl of each sample was visualised with the microscope EVOS®FL Auto Imaging System (Life Technologies - Thermo Fisher Scientific, MA, USA). Images were captured at 40X magnification under the fluorescent channel RFP (531/40 nm excitation; 593/40 nm emission) edited with phase contrast. Moreover, the rate of yeast membrane permeabilisation was evaluated in a microtiter plate on 100 μl of samples, prepared as described above, with the difference that yeast cells were treated with the PI dye and protein concentrations simultaneously. After 5 h, the fluorescence was measured in a VarioscanLUX plate reader (Thermo Fisher Scientific, MA, USA) at the maximal excitation (λEx) and maximum emission (λEm) wavelengths of 535 nm and 617 nm.

### 2.8 Haemolysis assay

The release of haemoglobin from defibrinated sheep erythrocytes caused by the presence of the protein was calculated following the protocol outlined by Shwaiki [16]. Briefly, a 4% red blood cell (Oxoid™, Oxoid Ireland c/o Fannin Healthcare, Ireland) solution was incubated for 1 h at 37 °C with 50, 100 and 200 μg/ml concentrations of the antifungal protein. Samples incubated with 0.1% Triton X-100 and PBS were used as positive and negative control, respectively. Afterwards, samples were centrifuged at 1000 g for 10 min, and the OD at 405 nm of the supernatant was measured. Results are expressed as a percentage of haemolysis, with 10% being the threshold in the data interpretation; if >10%, the protein was considered haemolytic, and if <10%, it was not haemolytic. The calculations were made using the measured absorbance values and the formula below:

$$\text{\% Haemolysis}\;=\;\frac{\left(A405proteintreatment)-(A405PBS\right)}{\left(A4050.1\text{\% }TritonX-100\right)-\left(A405PBS\right)}$$

### 2.9 Cytotoxicity assay

The cytotoxicity of the antifungal protein was tested by measuring cell viability using a MTT cell viability kit (Cell proliferation Kit I MTT, Sigma Aldrich, MO, USA). The protocol was performed as described by Shwaiki [16]. Human colonic cells, Caco-2 cells (ECACC) were maintained and passaged in Dulbecco's Modified Eagle Media (DMEM) supplemented with 1% non-essential amino acids and 10% Fetal Bovine Serum (FBS). A 200 μl cells inoculum (1 × 10^5^ cells/ml) was added into wells of a flat-bottom 96 well microtiter plate and incubated for 24 h at 37 °C with 5% CO_2_ allowing cells to reach confluence. After removing the media, the protein was added at concentrations of 100, 200, 300, 400, 500, 600 and 700 μg/ml in conjunction with DMEM and 2.5% FBS. Untreated cells served as positive control and wells without cells were used as negative control (0% viability), since the assay is based on the reduction of 3-[4,5- dimethylthiazol-2-yl]-2,5-diphenyltetrazolium bromide (MTT) by living cells. After 24 h incubation in the same conditions, the media was removed, and 100 μl of DMEM plus 10 μl of MTT labelling reagent (Cell proliferation Kit I MTT, Sigma Aldrich, MO, USA) were added to each well. A further 4 h incubation followed this step before adding 100 μl of solubilisation buffer. Next, the plate was incubated overnight, and the viability of the cells was measured using a fluorometric spectrophotometer at 570 nm with a background reading of 690 nm.

### 2.10 Stability tests

The protein's stability in high heat, high salt and a range of pHs was established to evaluate changes in its antifungal potency when exposed to different environmental conditions. Protein concentrations of 37.5 μg/ml (½ MIC), 75 μg/ml (MIC) and 150 μg/ml (2MIC) were tested against the yeast *Z. bailii*. To study the protein's thermal stability, a purified protein sample was heated for 15 min at 100 °C and left to cool for 30 min before testing with a growth curve assay as described in section 2.4. The stability of the protein in different salt solutions was tested using SD broth modified with magnesium chloride (MgCl_2_) and potassium chloride (KCl) at two different concentrations: 1 mM and 5 mm, and 50 mM and 150 mM, respectively. The antiyeast assay was carried out on *Z. bailii* in the presence of the four salt concentrations, and a control consisted of media containing the salts without the protein. Similarly, different ranges of pH were tested by a growth curve assay where the pH of SD broth was changed to 3, 5, 7, 9 and 11, using 1 M sodium hydroxide and 0.1 M hydrochloric acid. Controls consisted of regular SD pH-adjusted broth without added protein.

### 2.11 Resistance to proteolysis

The protein's resistance to proteolytic digestion was tested with α-chymotrypsin (Sigma Aldrich, MO, USA), a common digestive enzyme found in the human gut. The assay was carried out as described by Shwaiki [14]. Briefly, protein samples were incubated with α-chymotrypsin at different peptide: enzyme molar ratios of 60:1, 250:1, 2500:1, for 4 h at 37 °C. The α-chymotrypsin was then thermally inactivated at 80 °C for 10 min before a growth curve assay with protein concentrations of 37.5 μg/ml, 75 μg/ml and 150 μg/ml was performed against the yeast *Z. bailii*. The α-chymotrypsin was stored in solution in a digestion buffer consisting of 50 mM Tris–HCl (pH 7.4) and 5 mM calcium chloride (CaCl_2_).

### 2.12 Statistical analysis

All the tests were run in triplicate or quadruplicate and results reported in tables and graphs are presented as means ± standard deviation. Statistic tests were performed on the program Analysis ToolPak (Microsoft Excel). In general, the statistical significance of the difference between behaviours observed in samples with protein and controls without protein was calculated with student's t-test. A probability of p < 0.05 was considered statistically significant.

## 3 Results

### 3.1 Selective isolation of the antifungal protein

**Fig. 1:**
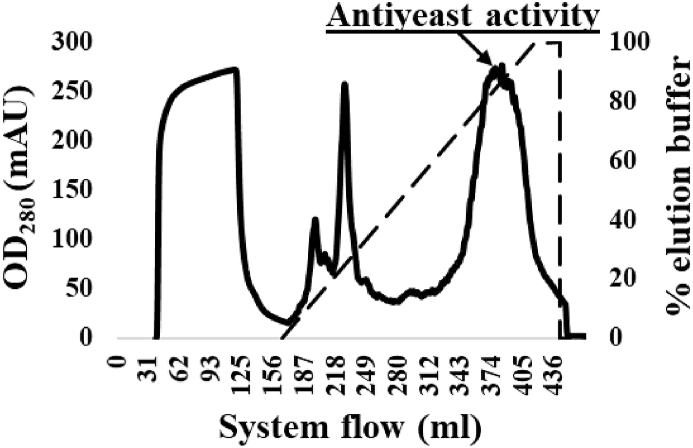
Separation chromatogram of one cycle of cation-exchange on an AKTA protein purification system from data directly exported from the program UNICORN. Antiyeast fractions were detected in the last peak (highlighted by the arrow).

Following the buffer extraction applied to white mustard seeds, the resultant proteins and peptide extract was submitted to chromatographic purification on an AKTA system, using one cycle of cation-exchange. After elution with 1 M NaCl, all the eluted fractions were tested for their antifungal activity against the type strain yeast *S. cerevisiae* DSM 70449 using a growth curve assay (data not shown). Fractions that presented killing activity were all located in the last peak of the chromatogram in Fig. 1; they were polled together, dialysed in 3.5 kDa cut-off tubing against distilled water overnight and freeze-dried. The resulting powder was dissolved in distilled water to reach 2 mg/ml concentration, filter-sterilised (pore size: 45 μm) and stored at −20 °C for further analysis. The protein concentration was determined by a BCA (Bicinchoninic Acid) assay for total protein quantification (QuantiPro BCA Assay Kit).

### 3.2 Gel electrophoresis

**Fig. 2:**
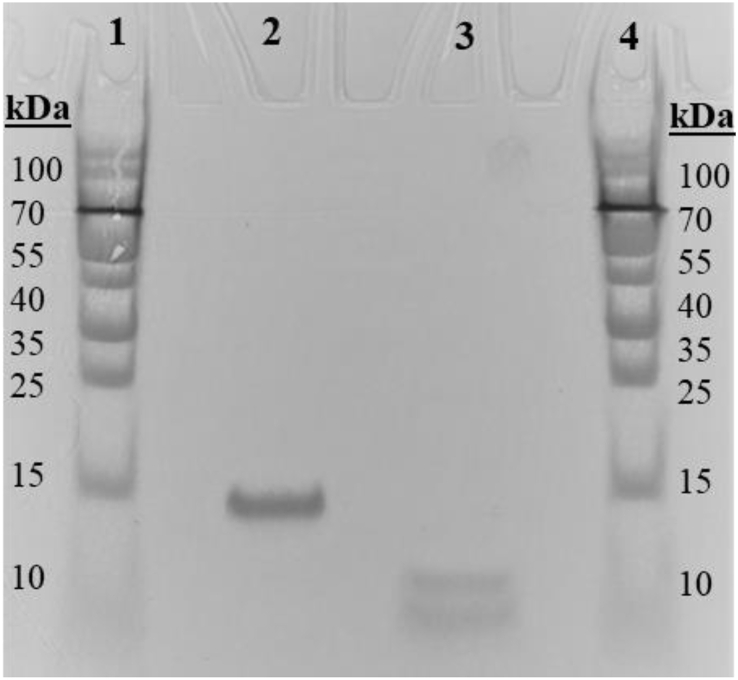
SDS PAGE with reduced and non-reduced purified protein samples. Lanes 1 and 4: molecular weight ladder; Lanes 2 and 3: non-reduced and DTT-reduced protein samples, respectively. The non-reduced sample appeared as a single band at the 14 kDa region, while the reduced sample appeared as two bands at the 9 and 5 kDa regions resulting from disruption of di-S bridges in the protein.

An SDS-PAGE gel was prepared to confirm that the protein isolation process had been carried out correctly. Non-reduced and reduced (with DTT) protein samples were prepared and loaded onto the gel; after the run, the non-reduced sample appeared as a single band (around 14 kDa), while the sample reduced with DTT appeared as two bands, one as 9/10 kDa and the second one at 5/4 kDa (Fig. 2). It can be concluded that the protein sample is pure; it contained only one type of protein of 14 kDa. Moreover, since DTT breaks down disulphide bonds that participate in a protein tertiary structure, the protein under investigation comprises two polypeptide chains held together by SS bonds.

### 3.3 Protein identification

**Fig. 3:**
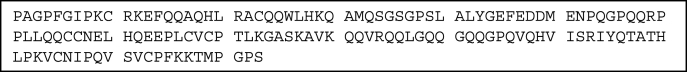
The amino acid sequence of the antifungal protein was proposed after the *de novo* sequence analysis. The sequence possesses a 96% identity with the sequence of the protein *Allergen Sin a 1*.

**Fig. 4:**
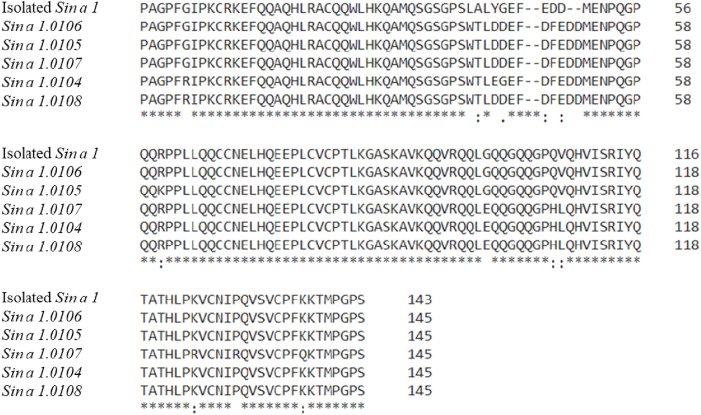
Cluster Omega alignment of the amino acid sequences of the isolated antifungal protein *Sin a 1* and the natural isoforms of *Allergen Sin a 1*: *Sin a 1.0106* (accession number: CAA62911.1), *Sin a 1.0105* (CAA62910.1), *Sin a 1.0107* (CAA62912.1), *Sin a 1.0104* (CAA62909.1) and *Sin a 1.0108* (CAA62908.1). Ninety % of the alignment results to be conserved amino acid positions (*), and cysteines residues are in an identical conserved position.

The protein sequencing by LC-MS/MS was carried out successfully, and the primary sequence of the antifungal protein is reported in Fig. 3. The sequence obtained was submitted to a BLASTp analysis (data not shown). All homologues resulted in members of the *Brassicaceae* family and belonged to *Sinapis*, *Brassica* or *Raphanus* genera. The protein with the highest sequence identity (96%) was *Allergen Sin a 1.0106* (NCBI accession number: CAA62911.1); a 2S (Storage Seeds) albumin member of the Napin/Bra allergen family. According to the literature [17], *Sin a 1* is composed of two different chains of 39 (small chain: 1–39) and 91 (large chain: 55–145) amino acid residues and a linker peptide (40–54) that is excised during the maturation process of the protein. An alignment was created on Clustal Omega server among the protein under investigation and all the other five natural isoforms of *Allergen Sin a* 1. This alignment was done to show the natural polymorphism of the protein *Sin a 1*. The six sequences alignment (Fig. 4) displayed a total of 90% of fully conserved amino acid positions, highlighted in Fig. 4 by the asterisk (*), while only 5% of not conserved positions, represented by a blank space. Moreover, the two functional chains (small and large) of *Allergen Sin a 1.0106* are composed of identical amino acid residues compared to the antifungal protein purified from mustard seeds; the only mutation present is recorded in the linker peptide. The antifungal protein under investigation is a natural isoform of the protein known as *Allergen Sin a 1*.

### 3.4 Minimum inhibitory concentration

*Sin a 1* possessed antifungal activity, but no antibacterial activity was recorded; results are summarised in Table 1. *Sin a 1* could completely inhibit the growth of all the yeasts and filamentous fungi tested with variable MIC values depending on the fungal strain. The variability in *Sin a 1* antifungal potency is evident when examining results for the yeast *S. cerevisiae*, where the three tested strains resulted in different MIC values. The industrial strain of Baker's yeast MTU 01P was sensitive only to the highest tested concentration of *Sin a 1* (1000 μg/ml), while the strains DSM 70449 and NCYC 77 were sensitive to smaller quantities of *Sin a 1* (100 or 200 μg/ml). The four spoilage yeast strains, *K. lactis*, *D. hansenii*, *Z. bailli* and *Z. rouxii*, were completely inhibited by 200–250 μg/ml, 150–200 μg/ml, 50–100 μg/ml and 400–500 μg/ml, respectively. In addition, the MIC value recorded for the pathogenic yeast *C. albicans* was 1000 μg/ml. Concerning the growth of filamentous fungi, *F. culmorum* and *A. fumigatus* were inhibited by *Sin a 1* at 50–100 μg/ml and 1000 μg/ml, respectively. Yeast strains *S. cerevisiae* DSM 70449 (type strain) and *Z. bailii* (spoilage strain) resulted as the most susceptible to the protein; thus, they were used to investigate *Sin a 1* antiyeast efficiency, mechanism of action and potency in different environmental conditions.

### 3.5 Colony count assay

**Fig. 5:**
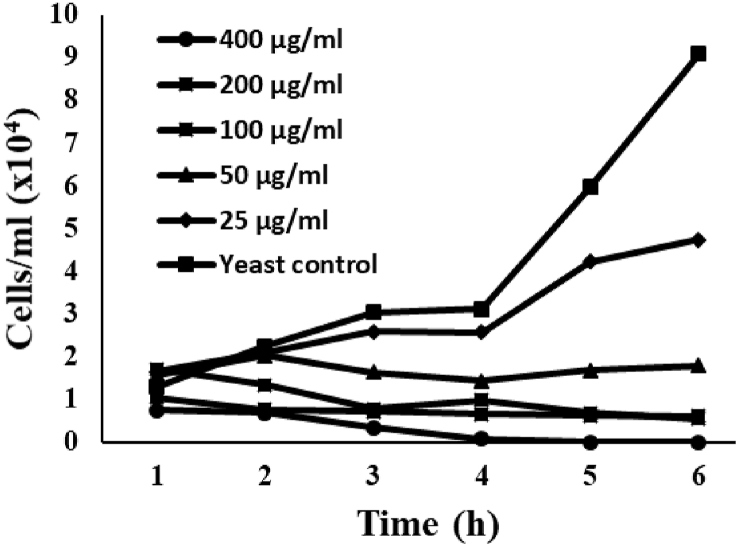
Yeast colony count assay demonstrating the rate of *S. cerevisiae* inhibition caused by *Sin a 1*. Yeast growth reduced after only 3 h of incubation at the highest protein concentrations compared to the control, which showed a steady increase in growth over the 6 h.

In the presence of *Sin a 1*, *S. cerevisiae* growth is always affected compared to the control with water that reached 9.08 × 10^4^ cells/ml after 6 h (Fig. 5). In the presence of the two smallest *Sin a 1* concentrations tested (25 and 50 μg/ml), *S. cerevisiae* growth resulted impacted; however, an increase in the growth was observed over time. A visible decrease in the growth was noted at the MIC and double MIC levels (100 and 200 μg/ml); however, the decrease was delayed compared to the highest concentration tested (400 μg/ml), where no yeast growth was recorded after 4 h. The killing efficiency of *Sin a 1* seemed to be expressed in a dose-dependent manner, with a faster decrease in the yeast growth at higher protein concentrations.

### 3.6 Total nucleotide leakage

**Fig. 6:**
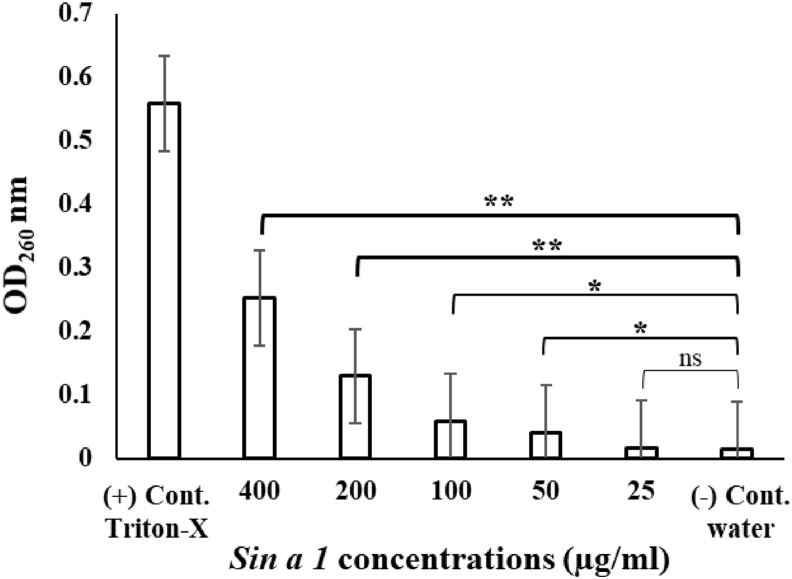
Degree of total nucleotide leakage caused by the presence of *Sin a 1* at different concentrations; increasing amounts of protein translated in greater nucleotide leaked from *S. cerevisiae* cells. The significance of the difference between protein samples and the negative control without protein was calculated with student's t-test (mean ± SD; n = 3; *p < 0.05, **p < 0.01, ***p < 0.001 and ns: no significant difference).

The amount of nucleotide leakage generated by cells of *S. cerevisiae* after a 5 h exposure to varying concentrations of *Sin a 1* was measured by spectrophotometry (Fig. 6). As expected, the highest OD at 260 nm recorded in this experiment was the 0.1% Triton-X control with a value of 0.559. Protein concentrations of 400, 200, 100 and 50 μg/ml produced a significantly higher OD (0.253, 0.130, 0.059 and 0.041, respectively) compared to the control with water (0.015). Moreover, these results suggested a dose-dependent correlation between the amount of antiyeast protein *Sin a 1* and the rate of yeast cells lysis.

### 3.7 Membrane permeabilisation

**Fig. 7:**
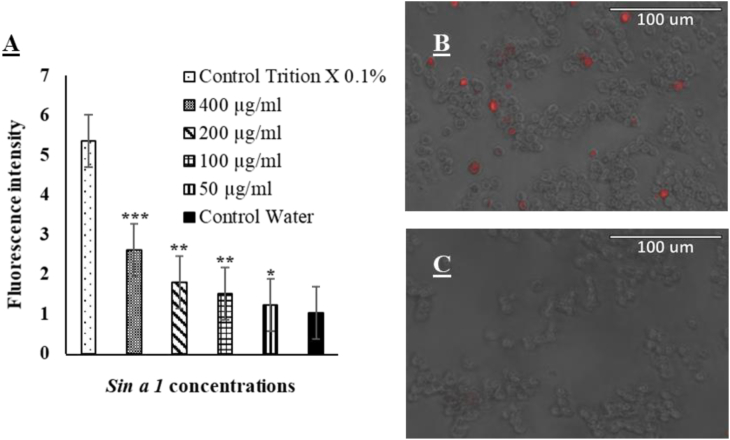
The impact of *Sin a 1* on *S. cerevisiae* cell membrane. Membrane disruption was implicated in all protein concentrations tested (A); the significant difference between protein samples and the control with water was established with student's t-test (mean ± SD; n = 3; *p < 0.05, **p < 0.01, ***p < 0.001 and ns: no significant difference). B: Red fluorescence from Propidium iodide dye indicates the presence of damaged cells in the 400 μg/ml sample compared to C, the control with water. (For interpretation of the references to colour in this figure legend, the reader is referred to the Web version of this article.)

In this assay, *Sin a 1*'s ability to induce membrane permeabilisation in *S. cerevisiae* was evaluated. In general, *Sin a 1* was found to cause damage in the yeast membrane; all the protein concentrations tested recorded a level of permeabilisation significantly higher than the control with dH_2_O. In addition, the rate of membrane permeabilisation decreased as the protein concentration was lowered, suggesting a positive correlation between the dose of *Sin a 1* and antiyeast effectiveness again. After 5 h exposure to 50, 100, 200 and 400 μg/ml of *Sin a 1*, yeast cells were treated with the florescent dye PI. The fluorescence emitted by cells subjected to membrane permeabilisation was visually observed with fluorescence microscopy (Fig. 7B and 7. C) and quantified by fluorescence spectroscopy (Fig. 7A). Excluding controls values, *S. cerevisiae* showed the highest fluorescence intensity (2.613) when treated with 400 μg/ml of protein. At the same time, in the 50 μg/ml sample, the fluorescence observed and recorded (1.229) was the lowest.

### 3.8 Haemolysis assay

This assay was carried out to observe the potential of 50, 100 and 200 μg/ml of *Sin a 1* to rupture mammalian red blood cells. According to the MIC results, these protein concentrations were lethal for several fungi, and yeast strains and this test was performed to characterise *Sin a 1* safety for mammalian cells. All the *Sin a 1* concentrations tested were not haemolytic (data not shown). The percentage of haemolysis was expressed by the haemolytic ratio (formula reported in Material and Method section), and no significant differences were found amongst the different concentrations of protein (50, 100, and 200 μg/ml resulted in 7.3%, 6.4% and 8.8%, respectively).

### 3.9 Cytotoxicity assay

**Fig. 8:**
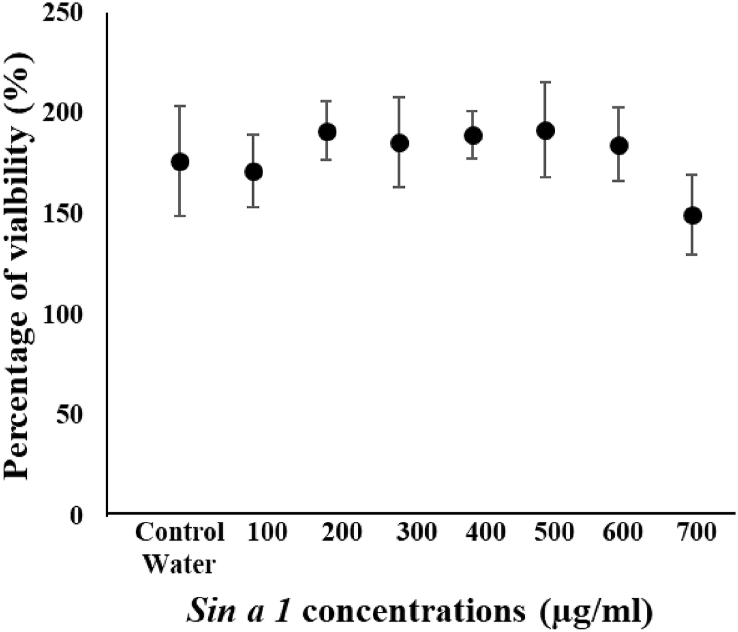
Human Caco-2 cell viability assay data after exposure to varying concentrations of *Sin a 1*. No significant variation in cell viability is evident compared to the control with water (untreated cells).

The cytotoxicity assay was carried out to characterise *Sin a 1* safety for human cells. The results indicated that the protein was not cytotoxic for Caco-2 cells at any concentrations tested. Moreover, no significant variance in the cell viability was noticed between *Sin a 1* treated cells and untreated cells (Fig. 8).

### 3.10 Stability tests

**Fig. 9:**
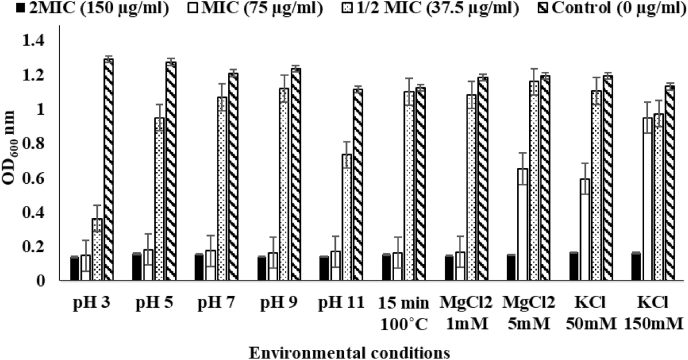
Demonstration of high stability of *Sin a 1*. The MIC for antiyeast activity against *Z. bailii* was maintained at all pHs tested and after heating to 100 °C. The MIC level was observed to have doubled in the presence of salts like 5 mM of MgCl_2_ and KCl.

The heat stability of *Sin a 1* was tested at 100 °C for 15 min and monitored to determine if its antiyeast activity against *Z. bailii* was retained following treatment, and no variations in the protein activity were recorded. The presence of 1 mM MgCl2 unaltered the protein's activity; however, in all the other salt conditions tested, *Sin a 1* activity showed an increase of 1-fold in the MIC against *Z. bailii*. Finally, the stability of the protein was unchanged by different ranges of pHs. Overall, *Sin a 1* killing activity was not affected by heat treatment or any pHs variation; its stability was only slightly altered by the presence of salts (Fig. 9).

### 3.11 Resistance to proteolysis

*Sin a 1*'s resistance to proteolytic digestion by α-chymotrypsin was tested at different molar ratios of peptide: enzyme (60:1, 250:1 and 2500:1). Similar results (data not shown) were recorded for all the molar concentrations under investigation: *Sin a 1* is resistant to proteolysis, but the digestion affected the protein antiyeast potential, with a one-fold reduction in the antiyeast activity against *Z. bailii*. Specifically, yeast growth was always inhibited at 150 μg/ml (2MIC) concentration of *Sin a 1*, meaning that the enzyme activity did not degrade the protein. While yeast growth was visible at 75 μg/ml (MIC) and 37.5 μg/ml (½MIC) concentration of *Sin a 1* in all the protein:enzyme molar ratios tested.

## 4 Discussion

Resistant fungal strains represent a rising concern in human health, food industries and agriculture. Therefore, there is a need to discover new antifungal molecules to be used as alternatives to the current commercially available antifungal drugs, preservatives and fungicides. The interest in small proteins and peptides from the plant's immune system (also known as plant AMPs) is increasing for pharmaceutical and biotechnological applications, owing to their diverse genetic origin and multiple modes of action that can limit the emergence of resistant strains.

In this study, a protocol design to target small, cationic and amphiphilic proteins with potential antimicrobial activity was applied on white mustard (*B. hirta*) seeds, and a ∼14 kDa protein with activity against *S. cerevisiae* was isolated. The isolation protocol applied was simple; the protein was selectively extracted with ammonium sulphate precipitation and purified with only one step of chromatographic separation (cation-exchange) on an FPLC system (AKTA start). Nano LC-MS-MS identified the primary sequence of the isolated protein, and bioinformatics analysis identified the isolated protein as a new isoform of the mustard Napin protein known as *Allergen Sin a 1*. After the different purification steps, an approximate amount of 1.2 mg of protein was recovered from 1 g of dry seeds. According to previous reports [18,19], *B. hirta* seed have a relatively high protein content (30%), and the protein/seed ratio for *Sin a 1* is assessed to be 0.82–2.94 mg/g depending on the type of mustard line.

In general, *Sin a* 1 has been mainly studied for its allergenic potential, and it is indeed considered a major allergen [20]. The primary biological function described for *Sin a 1* is nutrients reservoir for seedlings germination; however, several proteins belonging to the same family (2S albumins, also called Napins in *Brassicaceae*) have been studied for their antimicrobial *in vitro* properties [21] and defence against phytopathogens has been suggested as a secondary biological function. To our knowledge, this work reports the first description and characterisation of *Sin a 1*'s antimicrobial potential.

*Sin a 1*'s antimicrobial activity was investigated on 13 microbial strains, including two bacteria, two filamentous fungi, and nine yeasts. It exhibited antifungal and antiyeast properties, but no antibacterial activity was detected. Among the most sensitive strains, the type yeast *S. cerevisiae* DSM 70449, spoilage yeast *Z. bailii* Sa 1403 and the phytopathogen fungi *F. culmorum* FST 4.05 were the lowest MIC range reported (50–100 μg/ml or 3–6 μM). Results obtained seems coherent with data on the antifungal activity of other Napin proteins, *e.g.* the report from Thery [9] describe a Napin protein from broccoli seeds (*Brassica oleracea* var. *italica*) with a MIC of 37 μg/ml against *F. culmorum*; and the Napin protein from rapeseeds meal (*Brassica napus*) showed an IC_50_ value of 70 μM against the fungi *Fusarium langsethiae* [21]. The antiyeast activity of *Sin a 1* was further investigated utilising the two most sensitive yeasts strains, *S. cerevisiae* DSM 70449 and *Z. bailii* Sa 1403.

Napins are characterised by a compact heterodimeric structure composed of 40–50% of α-helices motifs and stabilised by four disulphide bridges linking eight conserved cysteine residues [22]. Like most plant AMPs, Napins are positively charged proteins, and they bear antimicrobial potential by accumulating at membrane surfaces via electrostatic interactions with the anionic phospholipids of the microbial membrane [23]. This interaction can mediate direct membrane disruption, leading to cytoplasmic leakage and cell death. The amphipathic α-helical structure of Napins and 2S albumins is thought to be the determinant of their ability to permeabilise fungal membrane [24], and it has been linked to the mechanism of CaM (calmodulin) antagonism [25].

Experimental findings suggested that *Sin a 1* mode of action involved yeast's membrane permeabilisation and caused leakage of cytoplasmatic components. Additionally, the membrane permeabilisation and cytoplasm displacement rate were positively correlated to the quantity of *Sin a 1* present. A colony count assay confirmed that *Sin a 1* antiyeast potency over time depended on the amount of protein, suggesting a dose-dependent relationship. In general, after 6 h of exposure, *Sin a 1* displayed the ability to influence and reduce the yeast growth even at not lethal concentrations (50 and 25 μg/ml); while a higher dose of the protein (400 μg/ml) had a faster action and was toxic after only 4 h.

Although the protein *Sin a 1* is recognised for causing IgE-mediated allergic reactions in sensitive individuals [20], the protein's safety was evaluated to judge its ability to cause direct harm to mammalian cells. A cytotoxicity assay against human CaCo2 cells and a haemolytic assay against sheep's red blood cells showed that, like other 2S albumins, *Sin a 1* is not harmful to mammalian cells at concentrations toxic for several fungal strains.

Non-cytotoxic AMPs with significant *in vitro* antimicrobial activity can still have limited biotechnological applications as they tend to lose activity when exposed to different environmental or physiological conditions, *e.g*. elevated salt concentration [26,27]; additionally, they can be susceptible to damage by proteases [28] which effects in poor or incomplete *in vivo* activity.

According to the available literature, *Sin a 1*'s compact structure causes the protein to resist heat degradation and trypsin proteolysis [29]. In general, trypsin-inhibition is described as a defensive mechanism used by plant compounds as protection against pests and pathogens that express proteinases [30] and is a feature shared among Napins [31,32]. Furthermore, the Napin protein from *Brassica napus* showed minimal structural changes at different pHs [33].

Experimental data confirmed the stable nature of *Sin a 1*. The protein was able to fully retain its activity against the yeast *Z. bailii* after heat treatment at 100 °C or when exposed to different ranges of pHs (3, 5, 7, 9 and 11). *Sin a 1*'s antiyeast activity was only slightly affected by salts; the presence of 5 μM of MgCl_2,_ 50 and 150 μM of KCl resulted in a one-fold loss in the MIC levels. A one-fold reduction in the antiyeast activity (MIC levels) against *Z. bailii* was recorded upon digestion by the protease α-chymotrypsin, confirming *Sin a 1*'s overall resistance to proteolytic digestion.

The antifungal potency, the non-toxic nature against mammalian cells and the structural stability of *Sin a 1* are all desirable features for possible biotechnological applications; however, in this case, the high structure stability translates to allergenicity potential, limiting the prospects for the use of this protein in its native form. *Sin a 1*'s exploitation as therapeutic or preservative is thus improbable; nevertheless, applications not involving human's consumption can be considered. *E. g.*, various AMPs have been expressed in plants to confer resistance towards phytopathogenic fungi, such as the hevein-like Pn-AMPs expressed in tobacco [34]. Amino acidic sequences of antimicrobial proteins can also be scanned for the rational design of small antimicrobial synthetic peptides [35]; therefore, despite being an allergenic protein, *Sin a 1* can promote the development of novel and beneficial antifungal molecules. This study confirms the antifungal potential of Napins from *Brassicaceae* and provides new insights on the biological functions of the protein *Allergen Sin a 1*.

## Credit author statements

**Giulia Mignone:** Conceptualization, Methodology, Validation, Investigation, Data Curation, Writing - Original Draft, Writing - Review & Editing.

**Laila N. Shwaiki**: Conceptualization, Methodology, Investigation, Resources.

**Elke K. Arendt:** Conceptualization, Resources, Supervision.

**Aidan Coffey:** Conceptualization, Review & Editing, Supervision, Project administration, Funding acquisition.

## Declaration of competing interest

The authors declare that they have no known competing financial interests or personal relationships that could have appeared to influence the work reported in this paper.

## Data Availability

Data will be made available on request.
